# 吸入伊洛前列素对肺动脉高压患者右心室功能改善的即刻效应：心脏MRI初步研究

**DOI:** 10.3779/j.issn.1009-3419.2015.03.07

**Published:** 2015-03-20

**Authors:** 青青 陆, 东 李, 振文 杨, 艳 韩, 倩 崔, 璋 张, 铁链 于

**Affiliations:** 1 300052 天津，天津医科大学总医院放射科 Department of Radiology, Tianjin Medical University General Hospital, Tianjin 300052, China; 2 300052 天津，天津医科大学总医院心内科 Department of Cardiovascular Disease, Tianjin Medical University General Hospital, Tianjin 300052, China

**Keywords:** 磁共振成像, 伊洛前列素, 高血压, 肺性, 心室功能，右, Magnetic resonance imaging, Iloprost, Hypertension, Pulmonary, Ventricular function, right

## Abstract

**背景与目的:**

肺动脉高压（pulmonary arterial hypertension, PAH）是以肺循环压力异常增高为特征的进展性疾病，可引起右心室（right ventricle, RV）功能进行性衰竭，最终导致死亡。因此RV功能的评估在PAH的诊断、随访中起着重要作用。心脏磁共振成像（cardiac magnetic resonance imaging, CMRI）成为无创评价心室功能的参照标准，尤其是RV功能。本研究通过CMRI评估吸入伊洛前列素对PAH患者RV功能影响的即刻效应。

**方法:**

2012年3月-2014年3月PAH患者48例，吸入单剂量20 μg的伊洛前列素溶液前、后立即进行CMRI检查，测量RV的舒张末期容积（end-diastolic volume, EDV）、收缩末期容积（end-systolic volume, ESV）、每搏输出量（stroke volume, SV）、射血分数（ejection fraction, EF）、心输出量（cardiac output, CO）、舒张末期面积（end-diastolic area, EDA）及收缩末期面积（end-systolic area, ESA）。RV面积变化百分比（percentage of RV area change, %RVAC）由公式[%RVAC=（EDA-ESA）/EDA×100%]计算获得。采用*Wilcoxon*符号秩和检验或配对t检验分析吸入伊洛前列素前、后RV功能参数变化。*P*＜0.05为差异有统计学意义。

**结果:**

吸入伊洛前列素后，患者的RV功能改善，RV EDV、RV ESV显著下降（*P*=0.007, *P*＜0.001），RV SV、RV EF及%RVAC增加（*P*=0.014, *P*=0.009, *P*=0.006），RV CO无变化（*P*=0.851）。

**结论:**

吸入伊洛前列素能立即明显改善PAH患者的RV功能，CMRI能准确、无创地评估该即刻效应。

## Abstract (en)

**Background and objective:**

Pulmonary arterial hypertension (PAH) is a progressive disorder characterized by abnormally elevated blood pressure of the pulmonary circulation. Without treatment, PAH progresses rapidly to right ventricular (RV) failure and even death. Cardiac magnetic resonance imaging (CMRI) has been an accurate and reproducible tool to assessment of RV morphology and function, which are important factors in the prognosis of patients with PAH. The aim of this study is to investigate acute RV response to inhalation of aerosolized iloprost in patients with PAH using CMRI.

**Method:**

From March 2012 to March 2014, 48 patients with PAH underwent CMRI before and immediately after inhalation of iloprost with a single dose of 20 μg over 15 min-20 min. RV function parameters derived from CMRI images were analyzed before and after iloprost inhalation, including end-diastolic volume (EDV), end-diastolic area (EDA), end-systolic volume (ESV), end-systolic area (ESA), stroke volume (SV), ejection fraction (EF) and cardiac output (CO). Percentage of RV area change was also calculated [%RVAC=(EDA-ESA)/EDA×100%]. *Wilcoxon's* Sign Rank Test or Paired Samples t-Test was used to compare the differences of RV function parameters before and after inhalation.

**Results:**

After iloprost inhalation, all patients showed significant decrease in RV EDV and RV ESV (*P*=0.007, *P* < 0.001 respectively). Whereas, there were significant increase in RV SV (*P*=0.014), RV EF (*P*=0.009) and %RVAC (*P*=0.006). RV CO had no significant difference before and after inhalation (*P*=0.851).

**Conclusions:**

Inhalation of iloprost can immediately improve RV function in patients with PAH, and noninvasive evaluation of the acute response with CMRI is feasibility.

肺动脉高压（pulmonary arterial hypertension, PAH）是以肺血管广泛重塑、阻力进行性增加而导致的肺循环压力异常增高为特征的进展性疾病，见于多种临床疾病，未经有效治疗或控制的PAH，可引起右心室（right ventricle, RV）功能进行性衰竭，最终导致死亡^[1]^。因此RV形态及功能的评估在PAH的诊断、随访、疗效评估和判断预后中起着关键作用。心脏磁共振成像（cardiac magnetic resonance imaging, CMRI）对RV有极佳的可视化能力和可重复性，对PAH的评估及随访有着独到的优势。吸入用伊洛前列素（iloprost，商品名Ventavis万他维）是一种选择性肺血管扩张剂，具有能明显改善PAH患者临床症状、降低心功能分级的潜力，目前被推荐为PAH重症患者的一线用药。本研究拟通过CMRI评估吸入伊洛前列素对PAH患者RV功能的即刻效应。

## 1 资料与方法

### 1.1 研究对象

2012年3月-2014年3月在天津医科大学总医院经右心导管（right heart catheterization, RHC）确诊的PAH患者48例，男性2例，女性46例；年龄12岁-65岁，平均（39.5±11.6）岁；心率（heart rate, HR）64 bpm-107 bpm，平均（81±10）bpm。PAH诊断标准为静息状态下由RHC测得的平均肺动脉压≥25 mmHg，肺动脉楔压≤15 mmHg，肺血管阻力 > 3 Wood units^[2]^。根据第五届世界肺高血压论坛分类^[3]^，48例PAH中，包括特发性肺动脉高压9例、未矫正先天性心脏病15例（房间隔缺损8例，室间隔缺损3例，动脉导管未闭4例）、结缔组织病24例（系统性红斑狼疮11例、混合型结缔组织病11例、干燥综合征2例）。通过病史、超声、肺动脉CT血管造影、肺功能等检查确定PAH的原因，并排除冠心病、心脏瓣膜病、慢性阻塞性肺疾病、慢性血栓栓塞性疾病等其他心肺疾病，排除肺静脉闭塞性疾病和肺毛细血管血管瘤病。

### 1.2 吸入方法

将吸入用伊洛前列素20 μg（万他维，德国拜耳先灵公司）与生理盐水2 mL混合后置入压缩空气式雾化吸入机（德国百瑞公司），嘱患者含住雾化吸入机口含器开始雾化吸入，持续缓慢深呼吸约15 min-20 min（平均17 min）。

### 1.3 图像采集

采用GE Twin-speed Infinity with ExciteII 1.5超导型MR仪（GE Healthcare, Milwaukee, WI, USA），8通道心脏相控阵线圈、心电门控技术、快速成像稳态采集序列（fast imaging employing steady-state acquisition, FIESTA）获得心脏短轴位图像。FIESTA序列短轴位成像参数：TR/TE min full/min full，翻转角45°，带宽125 kHz，FOV 35 cm×35 cm，矩阵224×224，扫描层厚8 mm，无层间隔，NEX 1。覆盖整个RV需10层-13层图像，每层扫描时相数为20，每层扫描时间为9 s-15 s，屏气。在吸入伊洛前列素前和吸入后立即进行相同参数的CMRI检查，两次检查时间共约40 min-50 min。

### 1.4 图像处理

将图像传输到AW4.3工作站（Advantage Windows version 4.3; GE Healthcare, Milwaukee, WI, USA），使用Report Card 3.7软件对图像进行后处理分析。分别以RV腔达最大容积与最小容积的时相作为舒张末期与收缩末期时相，并手动描记两时相图像中RV的心内膜轮廓（图 1）。RV容积包括RV流出道容积，节制索和小梁计入心室腔的部分。手动描记完成后软件自动计算RV的舒张末期容积（end-diastolic volume, EDV）、收缩末期容积（end-systolic volume, ESV）。每搏输出量（stroke volume, SV）为EDV与ESV的差值，射血分数（ejection fraction, EF）由公式（EF=SV/EDV^*^100%）计算获得，心输出量（cardiac output, CO）=SV^*^HR/1, 000。在RV中部层面获取RV的舒张末期面积（end-diastolic area, EDA）、收缩末期面积（end-systolic area, ESA），并由公式[%RVAC=（EDA-ESA）/EDA×100%]计算获得RV面积变化百分比（percentage of RV area change, %RVAC）。

**1:**
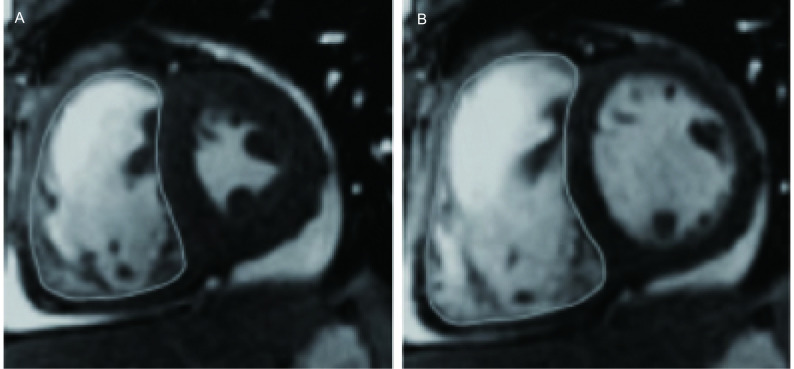
FIESTA心脏短轴位测量RV容积。A：收缩末期；B：舒张末期。图示描记RV心内膜轮廓。 Measurement of RV volume with short axis images of FIESTA. The endocardial borders of RV at end-systole phase (A) and end-diastole phase (B) were manually traced (the white arc line). FIESTA: fast imaging employing steady-state acquisition; RV: right ventricle.

### 1.5 统计学分析

采用SPSS 18.0统计软件。对测得的各项计量数据指标用*Shapiro*-*Wilk*检验进行正态分析，以均数±标准差（Mean±SD）或中位数（四分位间距）表示。符合正态分布的参数选用配对t检验进行分析，不符合正态分布的参数选用*Wilcoxon*符号秩和检验进行分析。*P*＜0.05为差异有统计学意义。

## 2 结果

吸入伊洛前列素前、后RV功能参数变化情况见表 1。吸入伊洛前列素后，RV EDV、RV ESV显著下降（*P*=0.007, *P*＜0.001），RV SV、RV EF及%RVAC显著增加（*P*=0.014, *P*=0.009, *P*=0.006），RV CO无显著变化（*P*=0.851）（图 2）。

**1:** 吸入伊洛前列素前、后PAH患者RV CMRI参数 RV function parameters in patients with PAH at baseline and immediately after iloprost inhalation assessed by CMRI

Parameters	Baseline	After inhalation	*Z* or *t*	*P*
RV EDV (mL)	190.8 (127.9)	186.3 (113.1)	*Z*=-2.646	0.007
RV ESV (mL)	127.3 (121.4)	122.7 (101.1)	*Z*=-4.954	< 0.001
RV SV (mL)	59.8 (26.0)	62.5 (32.7)	*Z*=-2.436	0.014
RV EF (%)	34.9±13.7	38.1±13.0	*t*=-5.757	0.009
RV CO (L/min)	4.7 (2.0)	4.7 (2.7)	*Z*=-0.195	0.851
RV EDA (cm^2^)	28.7±10.2	27.9±10.4	*t*=2.455	0.018
RV ESA (cm^2^)	23.5±10.2	22.1±10.2	*t*=4.598	< 0.001
%RVAC (%)	16.4 (17.3)	23.2±14.8	*Z*=-2.718	0.006
PAH: pulmonary arterial hypertension; EDV: end-diastolic volume; ESV: end-systolic volume; SV: stroke volume; EF: ejection fraction; CO: cardiac output; EDA: end-diastolic area; ESA: end-systolic area; %RVAC: percentage of RV area change. Variables with normal distribution were expressed as Mean±SD, and variables with skewness distribution were expressed as Md (Interquartile range, IQR).				

**2:**
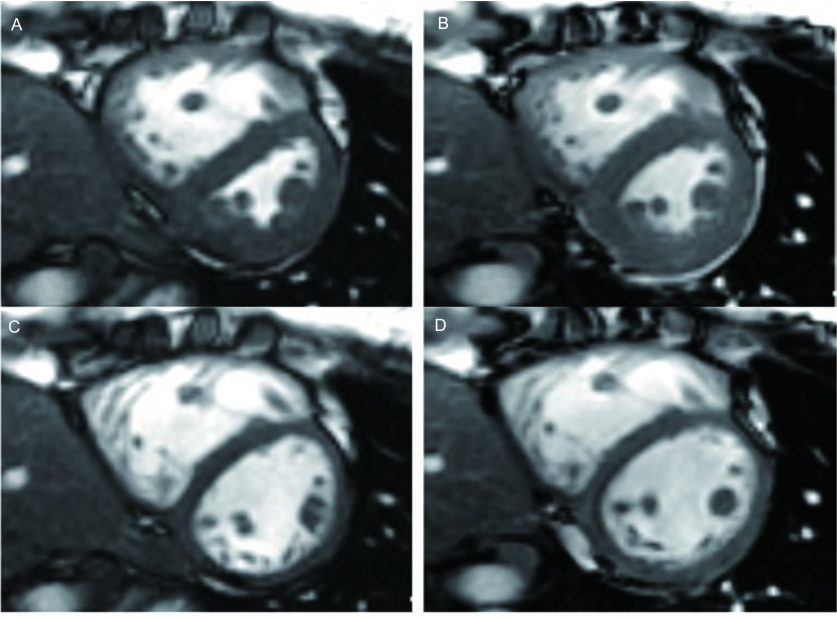
混合型结缔组织病并PAH，女，30岁，吸入伊洛前列素前、后FIESTA心脏短轴位图像比较。A、B分别为吸入伊洛前列素前、后心室中部收缩末期图像，C、D分别为吸入伊洛前列素前、后心室中部舒张末期图像。吸药后室间隔曲度较前好转，RV容积较前缩小，以收缩末期明显。 FIESTA short axis images before and after iloprost inhalation in a 30-year-old female PAH patient with mixed connective tissue disease. A, B show the mid-ventricular short-axis images at end-systole phase before and immediately after iloprost inhalation respectively; C, D are the corresponding images at end-diastole phase. After inhalation of iloprost, ventricular septal shifted toward the RV and there was significant reduction in RV volume, especially at end-systole phase.

## 3 讨论

目前，临床评估心功能的主要方法是超声心动图。然而，由于RV形态复杂，在短轴方位上呈新月形，在长轴方位上呈三角形，而且RV的收缩形式呈蠕动样，也与左心室不同，因此，超声心动图难以通过常规数学模型准确评估RV功能^[1]^。此外，超声心动图还可能因三尖瓣返流、肺内气体或其他肺部疾病的影响而使测量准确性大为降低。因此，超声心动图对PAH患者RV形态及功能的评估存在固有的局限性^[1]^。RHC检查能够测量肺动脉压力，评估相关血液动力学参数，是诊断PAH的金标准，但对RV形态和功能的评估亦存在缺陷。CMRI为临床提供出一种无创、简便、重复性高、准确性高的检查方法。CMRI的FIESTA序列为平衡稳态自由进动序列，具有良好的心肌与血池的对比度，能清晰地显示心肌的边界；成像速度快，一次屏气能获得20个以上时相数的图像，采用分段采集电影成像技术，能够生动地显示心脏的运动状态。该方法评估RV功能不需要解剖学假设，其三维的解剖学、形态学测量不受体型、肺疾病、胸壁畸形的影响，具有软组织分辨力高、对心脏解剖结构显示逼真的优势。CMRI成为无创评估心脏形态和功能的参照标准^[4]^，能够无创、准确地提供PAH患者RV的功能信息^[5-7]^。

本研究利用CMRI评估PAH患者RV功能，结果证实，吸入伊洛前列素能立即显著改善PAH患者RV功能，RV EDV、RV ESV较吸药前显著减小，RV SV、RV EF、%RVAC明显增加。这与Loureiro等^[8]^和Huez等^[9]^采用超声心动图的个案研究结果一致，他们观察到吸入伊洛前列素后RV容积立即明显减小、形态接近正常、RV功能改善。最近采用RHC评估PAH患者吸入伊洛前列素后主肺动脉血液动力学的急性改变的研究表明，吸药后肺动脉压和肺血管阻力显著下降^[10, 11]^。虽然RHC研究所观察的指标与本研究不同，但其反映的吸入伊洛前列素后血液动力学的快速改善与本研究一致。本研究中，吸入伊洛前列素后RV CO的变化不明显，与田庄等^[12]^的RHC研究结果一致，但也有文献^[11]^报道吸药后RV CO显著增加，但变化幅度不大[(3.7±1.7)L/min *vs* (3.9±1.9)L/min, *P*=0.009]。其原因可能与研究对象的严重程度及所处临床时期不同有关。

前列环素是花生四烯酸的代谢产物，由血管内皮细胞生成，具有扩血管、抗增殖及抗血小板聚集的生理作用。目前研究表明，PAH患者体内多种血管活性物质失衡，前列环素合成减少^[13]^。吸入用伊洛前列素是一种前列环素类似物，吸入后肺泡局部浓度较高，而毛细血管前阻力血管被肺泡所包绕，伊洛前列素可直接作用于毛细血管前阻力血管的括约肌和平滑肌细胞，因此具有肺血管选择性。伊洛前列素通过结合相关受体引起血管平滑肌细胞中的环磷酸腺苷（cyclic adenosine monophosphate, cAMP）升高，激活钙泵引起钙离子外流，同时打开钾通道，引起膜超极化；细胞内cAMP浓度升高还可抑制肌球蛋白激酶，有效抑制血小板聚集，最终引起血管扩张、血管阻力降低、血流量增加^[14]^。随着RV后负荷降低，RV容积减小，甚至可使CO增加。吸入20 μg/2 mL伊洛前列素溶液后，血药浓度峰值时间在吸入结束时至随后的5 min内，半衰期为21 min-25 min^[15]^，本研究中开始吸入伊洛前列素至CMRI检查的间隔时间约为15 min-20 min（平均17 min），能够有效地反映伊洛前列素对肺血管的即刻效应。

急性肺血管扩张试验是筛选对钙通道阻滞剂敏感的PAH患者的最可靠检查手段，在指导PAH患者治疗中具有重要意义。而目前该试验主要是在RHC检查过程中进行。本研究结果表明，CMRI能够监测RV功能的急性改变，且具有无创的优势，有望在监测PAH患者的急性肺血管试验中RV功能的变化起重要作用。

本研究患者吸入伊洛前列素前、后两次CMRI检查的扫描层面可能有轻微差异，但研究中观察的RV整体功能参数是通过各个层面所测容积积分所得，因此扫描层面的差异不会影响整体功能参数的计算。

吸入伊洛前列素能立即明显改善PAH患者的RV功能；CMRI能即刻准确、无创地评估RV功能变化，具有应用前景。
